# Study of thulium-167 cyclotron production: a potential medically-relevant radionuclide

**DOI:** 10.3389/fchem.2023.1288588

**Published:** 2023-10-19

**Authors:** Edoardo Renaldin, Gaia Dellepiane, Saverio Braccini, Alexander Sommerhalder, Hui Zhang, Nicholas P. van der Meulen, Robert Eichler, Zeynep Talip

**Affiliations:** ^1^ Center for Radiopharmaceutical Sciences (CRS), Paul Scherrer Institute, Villigen-PSI, Switzerland; ^2^ Department of Chemistry, Biochemistry and Pharmaceutical sciences (DCBP), University of Bern, Bern, Switzerland; ^3^ Albert Einstein Center for Fundamental Physics (AEC), Laboratory of High Energy Physics (LHEP), University of Bern, Bern, Switzerland; ^4^ Beam Physics, Proton Facilities, Accelerator Operation and Development, Large Research Facilities, Paul Scherrer Institute, Villigen-PSI, Switzerland; ^5^ Laboratory of Radiochemistry (LRC), Paul Scherrer Institute, Villigen-PSI, Switzerland

**Keywords:** cyclotron, thulium-167, auger electron, proton irradiation, cross sections, radionuclide production

## Abstract

**Introduction:** Targeted Radionuclide Therapy is used for the treatment of tumors in nuclear medicine, while sparing healthy tissues. Its application to cancer treatment is expanding. In particular, Auger-electron emitters potentially exhibit high efficacy in treating either small metastases or single tumor cells due to their short range in tissue. The aim of this paper is to study the feasibility of a large-scale production of thulium-167, an Auger-electron emitter radionuclide, in view of eventual systematic preclinical studies.

**Methods:** Proton-irradiated enriched erbium-167 and erbium-168 oxides were used to measure the production cross sections of thulium-165, thulium-166, thulium-167, and thulium-168 utilizing an 18-MeV medical cyclotron equipped with a Beam Transport Line (BTL) at the Bern medical cyclotron laboratory. The comparison between the experimental and the TENDL 2021 theoretical cross-section results were in good agreement. Additional experiments were performed to assess the production yields of thulium radioisotopes in the BTL. Thulium-167 production yield was also measured irradiating five different target materials (^167^ *Er*_2_ *O*_3_, ^168^ *Er*_2_ *O*_3_, ^*nat*^ *Tm*_2_ *O*_3_, ^*nat*^ *Yb*_2_ *O*_3_, ^171^ *Yb*_2_ *O*_3_) with proton beams up to 63 MeV at the Injector II cyclotron of Paul Scherrer Institute.

**Results and Discussion:** Our experiments showed that an 8-h irradiation of enriched ytterbium-171 oxide produced about 420 MBq of thulium-167 with a radionuclidic purity of 99.95% after 5 days of cooling time with a proton beam of about 53 MeV. Larger activities of thulium-167 can be achieved using enriched erbium-168 oxide with a 23-MeV proton beam, obtaining about 1 GBq after 8-h irradiation with a radionuclidic purity of < 99.5% 5 days post end of bombardment.

## 1 Introduction

For many years nuclear medicine has used radionuclides to diagnose and treat many diseases, including cancer, heart disease, neurological disorders, and dementia. Targeted Radionuclide Therapy (TRT) stands out as a nuclear medicine technique capable of targeting tumors of different sizes. This task is achieved by targeting molecules labeled with specific radionuclides. According to the radionuclide selected, a suitable particle radiation (*β* ^−^, *α* or Auger emission) can be adopted to deposit a cytotoxic radiation dose. The radionuclide is bound to a targeting agent, expressing an affinity towards tumor-associated antigens. The selection of a suitable targeting molecule favors the accumulation of the radiopharmaceutical preferentially in the proximity of malignancies, sparing healthy cells from excessive dose (Gudkov et al., 2016). In particular, radiopharmaceuticals labeled with Auger-electron emitters potentially exhibit a higher efficacy as compared to currently-employed *β* ^−^ emitters in eradicating micrometric tumors or single cells (Müller et al., 2019) due to higher linear energy transfer (LET), hence shorter range in tissue. In addition, recent studies have demonstrated that it is sufficient for Auger-electron emitters to be membrane-bound, without the necessity of being internalized to induce therapeutic effects (Borgna et al., 2022). However, the radiobiological effects induced by the deposited dose of Auger electrons still need to be further investigated. As a result, the urgency to produce high activities of radionuclides emitting Auger electrons can be justified in view of systematic preclinical investigations.

Recent research studies have focused on identifying a list of candidate radionuclides for TRT based on tumor-to-normal-tissue absorbed dose (Bernhardt et al., 2001; Uusijärvi et al., 2006), photon-to-electron energy ratio (Bernhardt et al., 2001; Filosofov et al., 2021), number of Auger electron emitted per decay (Filosofov et al., 2021). Thulium-167 is deemed as one of the promising candidates because of its appealing nuclear properties (e.g., *t*_1/2_ = 9.25 *d*, 100% electron capture (EC) decay) and significant emittance of low-energy electrons (Stepanek et al., 1996; Filosofov et al., 2021). In addition, its chemical behavior exhibits notable advantages in view of eventual radiolabeling of pharmaceuticals. Since lanthanides share similar coordination chemistry, a single delivering agent could be ideally labeled with several radiolanthanides according to the desired application. Furthermore, its effective diagnostic utility has been previously demonstrated when used as bone-scanning agent in metal or citrate forms detecting its intense gamma ray (I_*γ*,*abs*_ = 42%) of 208 keV (Chandra et al., 1971; Yano and Chu, 1975; Ando et al., 1983).

A comprehensive examination of existing literature about production cross sections for the thulium-167 production (Table 1) resulted in two experiments using natural (Tárkányi et al., 2008) and enriched erbium-167 (Tárkányi et al., 2010b) oxides as target materials. In addition, Dmitriev et al. (1980) previously investigated thulium-167 production using natural erbium oxide with low-energy (18 MeV–22 MeV) protons. Their experimental results reported a maximum production yield of 8.3 MBq/*μ*Ah. The same material was also irradiated with a 23-MeV proton beam at PSI for offline mass separation experiments at CERN-MEDICIS (Heinke et al., 2021). After an 8-h irradiation the highest produced activity was about 250 MBq.

**TABLE 1:** Table of investigated production routes of thulium-167, as either main or side product, present in the literature with nuclear reaction and investigated energy range. Data retrieved from EXFOR Library (Otuka et al., 2014).

Target	Reaction	Energy range (MeV)	Reference
^165^Ho	(*α*, 2*n*)	15.9–65.8	Mukhrjee et al. (1991); Martin and Pilger (1966); Gadkari et al. (1997); Tárkányi et al. (2010a); Usman et al. (2020)
^*nat*^Er	(*α*, *x*)	16.1–48.9	Király et al. (2008); Saito et al. (2019)
	(*d*, *x*)	4.6–49.6	Tárkányi et al. (2007a), Tárkányi et al. (2018); Uddin Khandaker et al. (2020)
	(*p*, *x*)	3.1–69.9	Rayudu and Yaffe (1963); Dmitriev et al. (1980); Tárkányi et al. (2008); Hermanne et al. (2011b)
^167^Er	(*d*, 2*n*)	9.7–20.7	Hermanne et al. (2011a)
	(*p*, *n*)	6.2–15.7	Tárkányi et al. (2010b)
^*nat*^Tm	(*α*, *α*2*n*)	37.1–46.8	Singh et al. (1990)
	(*α*, *x*)	38.4–65.8	Mohan Rao et al. (1992)
	(*d*, *x*)	14.4–49.8	Tárkányi et al. (2007b); Hermanne et al. (2009), Hermanne et al. (2016); Saito et al. (2017)
	(*n*, 3*n*)	15.1–30.5	Champine et al. (2016); Gooden et al. (2017)
	(*p*, *x*)	16.2–44.9	Tárkányi et al. (2012); Saito et al. (2020)
^*nat*^Yb	(*α*, *x*)	29.6–37.2	Tárkányi et al. (2017)
	(*d*, *x*)	22.0–48.2	Tárkányi et al. (2013), Tárkányi et al. (2014)
	(*p*, *x*)	11.2–68.8	Tárkányi et al. (2009)
^*nat*^Lu	(*p*, *x*)	590.0	Scholz et al. (1976)
^*nat*^Hf	(*p*, *x*)	590.0, 108.0–194.0	Scholz et al. (1976); Medvedev et al. (2008)
^*nat*^Ta	(*p*, *x*)	590, 195, 97–2580	Scholz et al. (1976); Michel et al. (2002); Medvedev et al. (2008)

The present paper focuses on two complementary objectives: i) the investigation of production cross sections of relevant thulium radionuclides using enriched erbium-167 and erbium-168 oxides as target materials with an 18-MeV medical cyclotron and ii) the investigation of different cyclotron production routes using a 72-MeV proton beam. In addition, the production yield results from the medical cyclotron were compared to those obtained using a 72-MeV research cyclotron (van der Meulen et al., 2020b). The following target materials were used: a) ^167^ *Er*_2_ *O*_3_, b) ^168^ *Er*_2_ *O*_3_, c) ^*nat*^ *Tm*_2_ *O*_3_, d) ^*nat*^ *Yb*_2_ *O*_3_, e) ^171^ *Yb*_2_ *O*_3_, mainly inducing the following nuclear reactions using proton beams with various energies:

a) ^167^ *Er* (*p*,*n*)^167^ *Tm*
b) ^168^ *Er* (*p*,2*n*)^167^ *Tm*
c) ^169^ *Tm* (*p*,3*n*)^167^ *Yb* →^167^ *Tm*
d) ^*nat*^ *Yb*(*p*,*xn*)^167^ *Lu* →^167^ *Yb* →^167^ *Tm*
e) ^171^ *Yb*(*p*,5*n*)^167^ *Lu* →^167^ *Yb* →^167^ *Tm*

## 2 Materials and methods

The two objectives of this study were achieved using two different cyclotrons. The cross-section measurements were performed at the Bern medical cyclotron laboratory utilizing an 18-MeV medical cyclotron (IBA Cyclone HC 18 MeV). The production yield measurements were conducted using a 72-MeV separated-sector high-intensity isochronous cyclotron, called Injector II, the second stage of the High-Intensity Proton Accelerator (HIPA), installed at PSI. Targets were prepared according to the requirements of each facility and irradiation purposes.

### 2.1 Target materials

All target materials consisted of oxide powder of different lanthanides. Target materials with natural isotopic composition were purchased from ChemPUR (Chem, 2023) with the following chemical purities: 1) 99.9999 %^*nat*^ *Yb*_2_ *O*_3_ and 2) 99.999 %^*nat*^ *Tm*_2_ *O*_3_. Isotopically enriched target materials were purchased from ISOFLEX United States (Isoflex, 2023) with the following enrichment levels: 1) ^167^ *Er*_2_ *O*_3_ 96.3%, 2) ^168^ *Er*_2_ *O*_3_ 97.2% and 98.3%, and 3) ^171^ *Yb*_2_ *O*_3_ 95.5%. Supplementary Table S2 and Supplementary Table S3 report the isotopic composition of each target material.

### 2.2 Cross-section and production yield measurements at the Bern medical cyclotron laboratory

**FIGURE 1:**
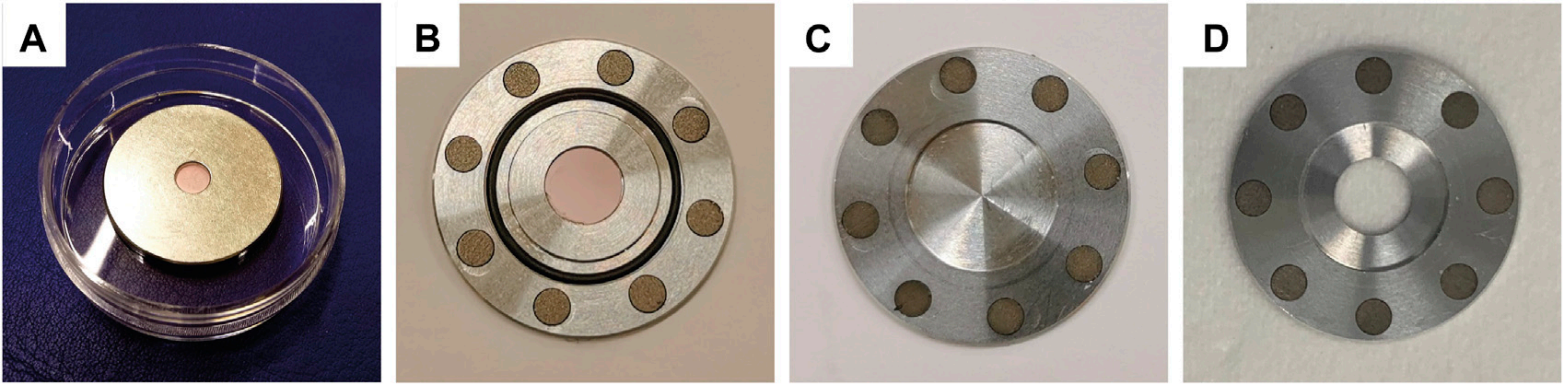
Targets used at the Bern medical cyclotron laboratory: **(A)** target holder for cross section measurements with sedimented erbium oxide, **(B)** target holder for production with an erbium oxide disk, **(C)** target lid, **(D)** target lid with central hole to reach the maximum proton energy.

Cross-section measurements were performed by means of a in-house custom-made target station (Dellepiane et al., 2021) installed at the end of a 6-m-long Beam Transport Line (BTL) (Braccini, 2013). The BTL brings the beam to a research-dedicated bunker with independent access. In this case, the targets for cross-section measurements consisted of aluminum disks with a central pocket (diameter 4.2 mm, depth 0.8 mm) (Figure 1A). Here, about 2 mg of the target material were deposited by means of a sedimentation technique to a thin layer (few micrometers) from a suspension of about 500 *μ*L of erbium oxide in ethanol. The target mass was gradually deposited pipetting about 5 *μ*L and waiting for the ethanol evaporation; the process was repeated until the entire central pocket was covered by the target mass. After deposition, the target mass was measured with an analytical balance (Mettler-Toledo, model: XSR205DU). Prior to irradiation, the disk was covered in 13-*μ*m thick aluminum foil to prevent radioactive material from spreading after the irradiation. Because of the low solubility of erbium oxide in ethanol, shaking the vial with the suspension before pipetting helped facilitating the material deposition over the whole pocket surface. However, a uniform surface distribution of the target mass is improbable using the sedimentation technique. For this reason, the cross-section measurements were performed with a technique developed by the Laboratory of High Energy Physics (LHEP) group (Carzaniga et al., 2017). Each target mass was fully bombarded with low proton currents ( <10 nA) (Auger et al., 2015) and with a flattened, collimated beam for short time periods (5 min–15 min). This method ensured a flat proton beam onto the target surface, allowing one to neglect the non-uniformity of surface mass distribution of the target material. The proton energy directed into the BTL was assessed with various independent methods resulting in (18.3 ± 0.4) MeV (Nesteruk et al., 2018; Nesteruk et al., 2019; Campajola et al., 2019; Häffner et al., 2019). The proton beam energy degradation was performed with stacked disks of aluminum with different thicknesses and each proton energy impinging on the target was calculated by means of a Monte Carlo code (SRIM (Ziegler et al., 2010)). The beam flatness was measured online with a beam diagnostic element named UniBEaM (Auger et al., 2017). The target station equipment also allows to measure the integrated charge hitting the target and it is connected to a negative bias voltage to repel secondary electrons. The activity produced was assessed by means of gamma-ray spectrometry with a High-purity Germanium (HPGe) (Canberra GR 2009) detector, calibrated in efficiency with a certified multi-peak gamma source. The net peak areas of the gamma spectra were fitted using a software for spectrum analysis: *InterSpec* (Chan et al., 2018). Each measurement was performed with a dead time smaller than 1%. The nuclear data of the relevant thulium radioisotopes used to assess the cross sections are reported in Table 2. Then, the experimental results were compared with the theoretical calculations obtained from TENDL 2021 library (Koning et al., 2019). The EoB activity was assessed using the following equation:

$$A\left(t_{\mathrm{EoB}}\right)=\frac{C_{det}}{\varepsilon_{det}\cdot I_{\gamma }}\cdot \frac{t_{RT}}{t_{LT}}\cdot \frac{\lambda e^{\lambda t_{c}}}{1-e^{-\lambda t_{RT}}}$$ (1)

where *C*_det_ is the net area of the full-energy peak of the main gamma line selected, *ɛ*_det_ the detector efficiency, *I*_*γ*_ the absolute branching ratio, *λ* the decay constant, *t*_*LT*_ and *t*_*RT*_ are, respectively, the live time and the real time of the gamma measurement. The cross sections are assessed by means of the following equation:

$$\sigma =A\left(t_{\mathrm{EoB}}\right)\cdot \frac{q}{dI/dA}\cdot \frac{m_{\mathrm{mol}}}{m\cdot N_{Av}\cdot \epsilon \eta }\cdot \frac{1}{\left(1-e^{-\lambda t_{i}}\right)}$$ (2)

where *q* is the elementary charge, *dI*/*dA* is the surface integrated current density, *A* the surface target area, *m* the mass of the irradiated target, *N*_*Av*_ the Avogadro’s number, *m*_*mol*_ the molecular mass of the target material, *ϵ* the isotopic abundance or enrichment level, *η* the stoichiometric number of the target atom and *t*_*i*_ the irradiation time. Considering the cross-section measurement method employed, the surface integrated current density can be approximated as follows: *dI*/*dA* ≈ *I*/*A*, because the beam profile is on average flat onto the target surface.

**TABLE 2:** Nuclear decay data (decay mode, half-life (*t*_1/2_), energy of the most intense gamma ray (*E*_*γ*_), absolute gamma-ray intensity (*I*_*γ*,*abs*_)) of the most relevant thulium radioisotopes used to identify the radionuclides with gamma-ray spectrometry. All the data obtained from IAEA Live Chart of Nuclides (Verpelli and Abriola, 2011) based on *Nuclear Data Sheets* (Baglin, 2000; Jain et al., 2006; Baglin, 2008; Baglin, 2010).

	^165^Tm	^166^Tm	^167^Tm	^168^Tm
**Decay mode**	EC 100%	EC 100%	EC 100%	EC 99.99% *β* ^−^ 0.01%
*t*_1/2_	30.06 h	7.70 h	9.25 d	93.1 d
**Main** *E*_*γ*_ **(keV)**	242.9	778.8	207.8	198.3
*I*_*γ*,*abs*_ (%)	35.5 (17)	19.1 (12)	42.0 (80)	54.5 (2)

To validate the cross sections measured, the production yield of each radionuclide was assessed by irradiating thick targets (thickness ≈270μm , shown in Figure 1B) in the BTL, using the same target station employed for cross-section measurements. Since the station is not equipped with any cooling system, it was necessary to limit the irradiation time (13–18 min) and proton current ( <10 nA). Two targets for each erbium oxide were irradiated. By employing this approach, it was also possible to prevent saturation effects when performing gamma-ray spectrometry and to measure the short-lived radionuclides, specifically thulium-165 and thulium-166. The BTL set-up allowed to provide a good control over the proton current and the beam profile. In this way, it was possible to derive the production yield for each relevant thulium radioisotope experimentally. The targets were prepared at PSI. Each target was manufactured by compressing 40 mg of the oxide powder into disks using a two-ton hydraulic press. The targets were tightly sealed into an aluminum holder, designed in-house, composed of two parts: a target holder (Figure 1B) and a lid. The latter was available into two configurations: a) 240-*μ*m thick aluminum lid (Figure 1C) and b) a lid with a central hole to exploit the maximum proton beam energy (Figure 1D). The eventual release of gaseous by-products or molten target material was prevented using a locking mechanism made of a constellation of permanent magnets and an o-ring. The whole target coin was also wrapped in aluminum foil only when using the lid with the hole.

### 2.3 Production yield measurements at Paul Scherrer Institute

Thulium-167 production was investigated using five different target materials: a) ^167^ *Er*_2_ *O*_3_, b) ^168^ *Er*_2_ *O*_3_, c) ^*nat*^ *Tm*_2_ *O*_3_, d) ^*nat*^ *Yb*_2_ *O*_3_, e) ^171^ *Yb*_2_ *O*_3_. The authors explored direct (Section 1, Reactions a) and b)) and indirect (Section 1, Reactions c), d) and e)) production routes using natural and enriched materials, aiming to maximize the final activity and minimize the radionuclidic impurities. Hence, it is crucial to address special consideration about some relevant thulium radioisotopes (Table 2). They can be divided into i) relatively short-lived, thulium-165 and thulium-166 with an half-life of 30.06 h and 7.70 h, respectively, and ii) long-lived, thulium-168, *t*_1/2_ = 93.1 d. After the irradiation, the content of short-lived radionuclides can be reduced by waiting for a certain time period, the *cooling time*, before starting the chemical separation. Since thulium-166 decays faster than thulium-165, the latter is the most hostile, thereby affecting the cooling time. On the other hand, thulium-168 content can only be minimized by selecting appropriate target material and proton beam energy. Therefore, special attention must be paid to the minimization of thulium-165 and thulium-168 contents, because they worsen the *radionuclidic purity (P*) by following the intended product through the chemical separation. This quantity is defined as the fractional activity of the target radionuclide with respect to the total activity of the sample.

A 50-*μ*A current is peeled off from the main beam of 2.5 mA delivered by Injector II. Behind the beam splitter, the proton beam is directed towards an irradiation station named IP2 (van der Meulen et al., 2020a; Grundler et al., 2020). The proton beam energy is degraded by means of cooling water and niobium foils. Their thickness varies from 1.0 to 3.5 mm, providing a proton energy ranging from 8.9 MeV to 34.0 MeV (Supplementary Figure S1A). Without any niobium foil, various cooling water thicknesses are capable of degrading the proton beam providing a proton energy range from 40.4 MeV to 62.6 MeV (Supplementary Figure S1B). Proton beam relevant characteristics are tabulated in Supplementary Table S1.

**FIGURE 2:**
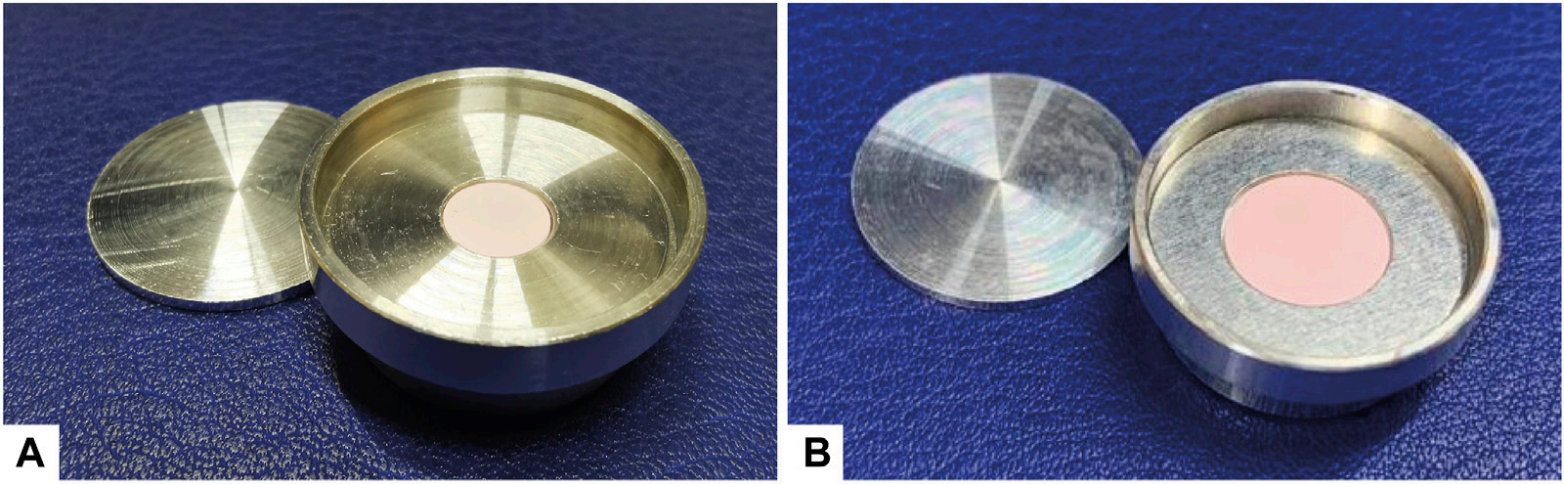
Target holders for production tests used at PSI: **(A)** target holder with a 6-mm-diameter disk of erbium oxide, **(B)** target holder with a 10-mm-diameter disk of erbium oxide.

Target diameter and mass are two closely related parameters for two reasons: i) mechanical stability of the target disk and ii) potential undesired production of radionuclidic impurities. From experimental evidence it was observed that the target disk is fragile, if the thickness is less than 250 *μ*m. This parameter also affects the exiting energy of the proton beam. As a result, careful attention should be focused on this parameter to minimize the production of undesired radionuclidic impurities, if applicable. Therefore, targets of 6 or 10 mm in diameter were prepared with 40 or 100 mg of mass, accordingly, to study the production yield. These configurations guaranteed to obtain mechanically stable target disks, whose mean thickness is about 270 *μ*m. Figures 2A,B illustrate the two configurations together with a 1-mm-thick lid and a target holder, both made of aluminum. The targets were prepared as explained in Subsection 2.2.

An irradiation procedure was established in order to validate the experimental and theoretical cross sections reported in the literature. In general, three energies covering the expected highest production cross-section peak of thulium-167 were selected for each target material. At least two targets of 40-mg mass were irradiated for each energy. The proton currents impinging on target surface were calculated with the program Beam Delivery Simulation (BDSIM) (Nevay et al., 2020) and TRANSPORT for the particle transport along the IP2 beam line. Once the optimum proton beam energy to obtain maximal thulium-167 activity was determined, additional experiments with 100-mg targets were conducted using the same energy only for enriched erbium oxides. The first irradiations were performed for short periods ( <30 minutes) in order to assess the produced side products at end of bombardment (EoB). This methodology was adopted to avoid high activities that would inevitably saturate the gamma detector. As a result, longer cooling times would have to be implemented, preventing detection of the short-lived radionuclides. Despite this expedient, it was mostly impractical to measure short-lived radionuclides (*t*_1/2_ < 1 day). Afterward, the irradiation time was increased to 2 h and, subsequently, to 8 h for the most promising production routes.

The activity was measured by means of gamma-ray spectrometry with a HPGe type *n* detector (model: EGC 07R, Intertechnique, France) connected to a data acquisition system from Canberra (Mirion^®^) (France) using their Genie 2000 software package for spectrometry analysis (RRID:SCR_021933). Each measurement was performed with a dead time <5% . The efficiency calibration was performed with a certified source of europium-152. Each result was corrected by the decay factor to obtain the EoB activity.

### 2.4 Uncertainty budget

Several independent contributions affect the cross-section uncertainties: target mass (<1%) ; integrated current (1%); beam flatness (5%); detection efficiency (<10%) , statistical error due to radioactive decay (<5%) ; uncertainties of nuclear properties (*u* (*t*_1/2_) ≈ 0.2%, *u* (*I*_*γ*_) ≈ 5%, in particular Iabs,γ(Tm167)=19% ). The overall cross-section uncertainty was calculated using the Gauss’ error propagation formula considering 1-*σ* level of confidence.

## 3 Experimental results

### 3.1 Characterization of target material

Enrichment levels of enriched target materials were assessed by means of inductively coupled-plasma mass spectrometry (ICP-MS) in collaboration with the Laboratory of Radiochemistry of Politecnico di Milano. The results are tabulated in Supplementary Table S2, S3 for enriched erbium oxides and ytterbium oxides, respectively, together with the data of the certificate of analysis provided by the manufacturer (ISOFLEX).

### 3.2 Cross section and production yield measurements at the Bern medical cyclotron laboratory

#### 3.2.1 Cross section measurements

**FIGURE 3:**
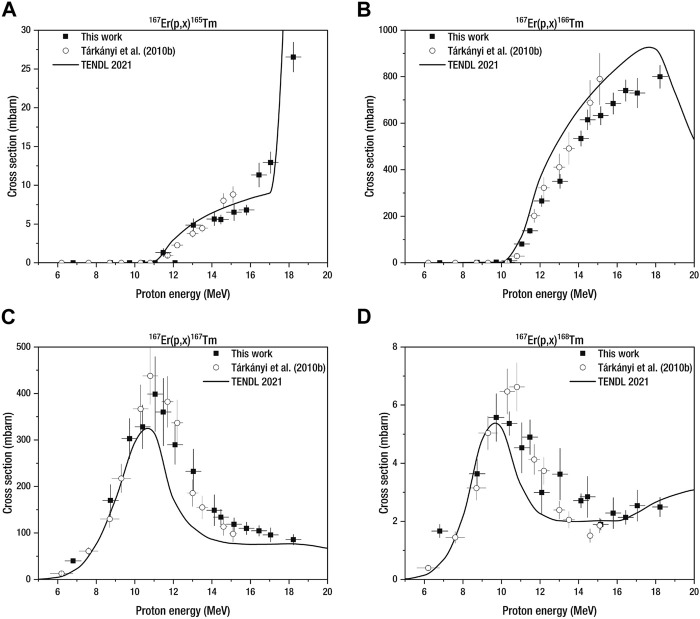
Comparison of experimental and theoretical production cross sections of **(A)** ^(165)^Tm, **(B)** ^(166)^Tm, **(C)** ^(167)^Tm, and **(D)** ^(168)^Tm in proton-irradiated enriched erbium-167 oxide. The results of Tárkányi et al. (2010b) are also reported, weighed by the enrichment level of our target material. The notation (*p*, *x*) includes (*p*, *γ*) and (*p*, *xn*) reactions, where *x* = 1, 2, . ., *n*.

**FIGURE 4:**
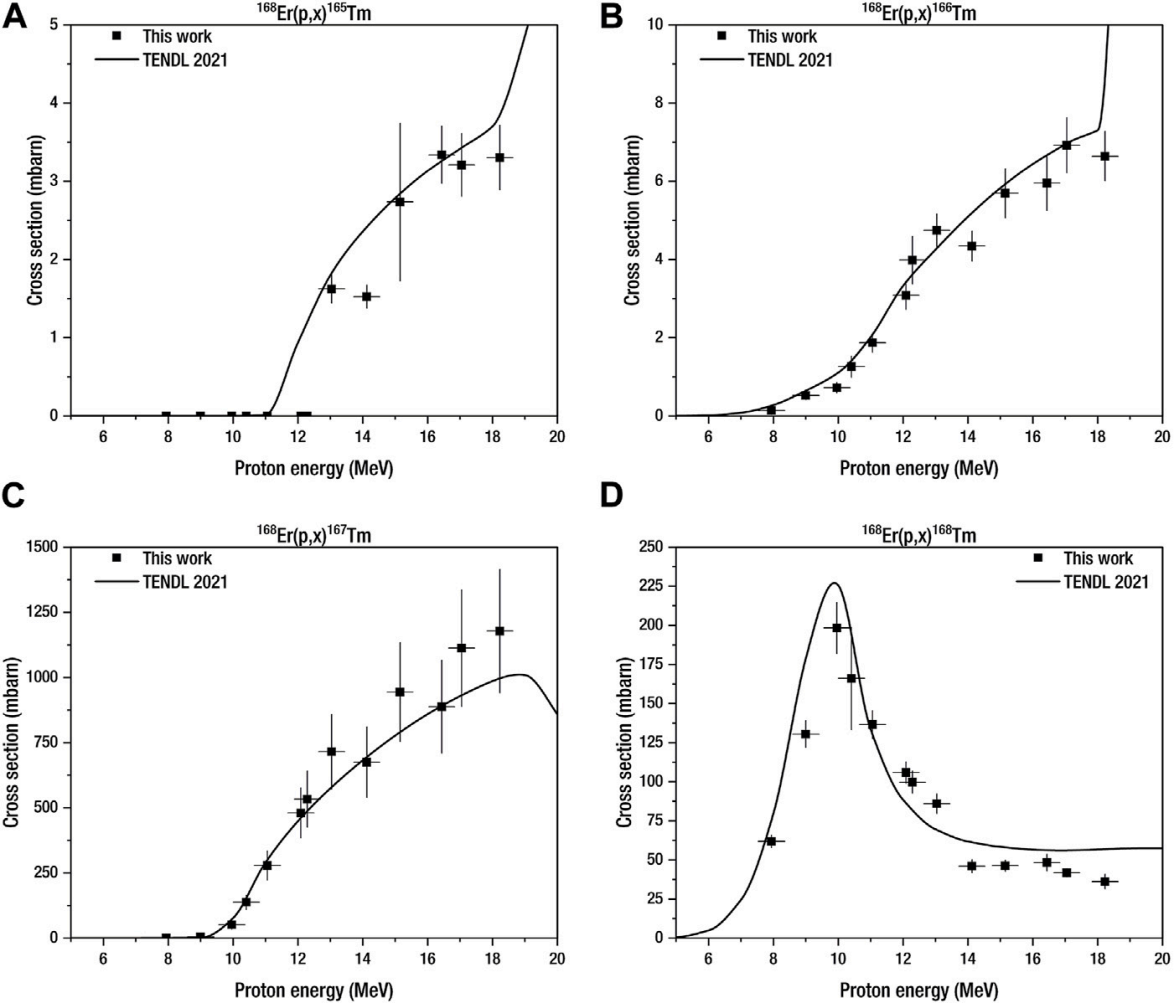
Comparison of experimental and theoretical production cross sections of **(A)** ^(165)^Tm, **(B)** ^(166)^Tm, **(C)** ^(167)^Tm, and **(D)** ^(168)^Tm in proton-irradiated enriched erbium-168 oxide. The notation (*p*, *x*) includes (*p*, *γ*) and (*p*, *xn*) reactions, where *x* = 1, 2, . ., *n*.

The production cross sections were investigated from 6.5 MeV to 18.2 MeV proton beam energy. The excitation functions of theoretical production cross sections and the experimental data are shown in Figure 3 and Figure 4 for ^167^Er_2_O_3_ and ^168^Er_2_O_3_, respectively. The cross-section results of Tárkányi et al. (2010b) are also plotted in Figure 3. The numerical cross-section data are reported in Table 3 and Table 4 for both target materials.

**TABLE 3:** Numerical data of production cross sections for the relevant thulium isotopes from proton-irradiated^167^Er_2_O_3_ material.

Energy (MeV)	^165^Tm (mbarn)	^166^Tm (mbarn)	^167^Tm (mbarn)	^168^Tm (mbarn)
6.8 ± 0.4			40.0 ± 4.9	1.67 ± 0.23
8.7 ± 0.4		1.26 ± 0.11	170 ± 34	3.64 ± 0.51
9.7 ± 0.4		2.39 ± 0.30	303 ± 43	5.57 ± 0.82
10.4 ± 0.4		8.36 ± 1.00	328 ± 47	5.36 ± 0.41
11.1 ± 0.4		80.4 ± 7.0	399 ± 80	4.53 ± 0.86
11.5 ± 0.4	1.31 ± 0.53	138 ± 12	360 ± 73	4.89 ± 0.60
12.1 ± 0.4	Not detected	266 ± 24	290 ± 42	2.99 ± 0.73
13.0 ± 0.4	4.87 ± 0.83	350 ± 30	233 ± 48	3.63 ± 0.89
14.1 ± 0.4	5.65 ± 0.84	534 ± 33	149 ± 33	2.71 ± 0.24
14.5 ± 0.4	5.59 ± 0.59	614 ± 43	134 ± 17	2.84 ± 0.71
15.2 ± 0.4	6.53 ± 1.09	632 ± 39	119 ± 14	1.89 ± 0.19
15.8 ± 0.4	6.81 ± 0.66	684 ± 46	110 ± 13	2.28 ± 0.53
16.5 ± 0.4	11.3 ± 1.5	740 ± 46	105 ± 11	2.14 ± 0.24
17.1 ± 0.4	12.9 ± 1.4	729 ± 63	95.9 ± 15.1	2.54 ± 0.53
18.2 ± 0.4	26.5 ± 1.9	800 ± 50	85.7 ± 11.1	2.49 ± 0.33

**TABLE 4:** Numerical data of production cross sections for the relevant thulium isotopes from proton-irradiated^168^Er_2_O_3_ material.

Energy (MeV)	^165^Tm (mbarn)	^166^Tm (mbarn)	^167^Tm (mbarn)	^168^Tm (mbarn)
7.9 ± 0.4		0.138 ± 0.054		61.8 ± 4.1
9.0 ± 0.4		0.520 ± 0.091	4.48 ± 0.93	130 ± 9
10.0 ± 0.4		0.722 ± 0.134	51.04 ± 10.42	198 ± 16
10.4 ± 0.4		1.26 ± 0.27	138 ± 28	166 ± 33
11.1 ± 0.4		1.87 ± 0.25	279 ± 56	137 ± 9
12.1 ± 0.4		3.09 ± 0.37	480 ± 97	106 ± 7
12.3 ± 0.4		3.99 ± 0.61	533 ± 107	99.7 ± 7.1
13.0 ± 0.4	1.62 ± 0.18	4.75 ± 0.42	715 ± 144	86.0 ± 6.2
14.1 ± 0.4	1.53 ± 0.15	4.34 ± 0.39	675 ± 136	46.0 ± 4.3
15.2 ± 0.4	2.73 ± 1.01	5.69 ± 0.63	944 ± 190	46.4 ± 3.6
16.4 ± 0.4	3.34 ± 0.37	5.95 ± 0.70	888 ± 178	48.3 ± 5.4
17.1 ± 0.4	3.21 ± 0.40	6.92 ± 0.71	1112 ± 224	41.8 ± 2.7
18.2 ± 0.4	3.31 ± 0.41	6.64 ± 0.63	1178 ± 237	36.2 ± 4.6

##### 3.2.1.1 Enriched erbium-167 oxide

^167^ *Er* (*p*,*x*)^165^ *Tm*: Experimental data and theoretical calculation are in good agreement (Figure 3A). The main contribution up to 17 MeV should be due to the ^166^ *Er* (*p*, 2*n*) reaction. It was also possible to experimentally observe the steep raise due to the ^167^ *Er* (*p*, 3*n*) reaction in the high energy range. Thulium-165 activity was measured at least 2 days after EoB to let thulium-166 decay due to an overlap between the main gamma line of thulium-165 (*E*_*γ*_ = 242.9 *keV*) and a coincidence sum peak between the most intense X-ray (48.2 keV, *I*_*abs*_ = 28% of K-L_2_ line) of thulium and a gamma line of 194.7 keV (*I*_*abs*_ = 0.83%) that originated from the thulium-166 decay. It is noteworthy that a considerable amount of thulium-166 was produced at the selected irradiation conditions using enriched erbium-167 oxide as target material.

^167^ *Er* (*p*,*x*)^166^ *Tm*: Data points and TENDL 2021 curve show a similar trend (Figure 3B). In this case, the main contribution should be assigned to the ^167^ *Er* (*p*, 2*n*) reaction.

^167^ *Er* (*p*,*x*)^167^ *Tm*: According to Figure 3C, experimental and theoretical data are in fairly good agreement. However, the TENDL 2021 prediction significantly underestimates the cross section between 11 and 16 MeV. Our experimental results confirm the results of Tárkányi et al. (2010b).

^167^ *Er* (*p*,*x*)^168^ *Tm*: Experimental data across the peak in Figure 3D are generally well described by theoretical calculations. However, as proton energy increases, the experimental trend is less consistent. At low proton energies the contribution of the ^168^ *Er* (*p*, *n*) reaction appears to be evident. In contrast, it is uncertain whether the slight increase at higher proton energies could be attributed either to the ^170^ *Er* (*p*, 3*n*) reaction or the error attributed to the measurement method.

##### 3.2.1.2 Enriched erbium-168 oxide

^168^ *Er* (*p*,*x*)^165^ *Tm*: In Figure 4A, the TENDL 2021 trend describes the experimental points well. The ^166^ *Er* (*p*, 2*n*) reaction likely dominates in this production route. In this case, it is worth mentioning that thulium-166 is produced in considerably lower activity. As a result, no significant cooling time was required.

^168^ *Er* (*p*,*x*)^166^ *Tm*: Figure 4B shows a good agreement between the theoretical excitation function and the experimental data. The production of this product also seems to be only influenced by the ^167^ *Er* (*p*, 2*n*) reaction.

^168^ *Er* (*p*,*x*)^167^ *Tm*: The theoretical calculations and the experimental data are in good agreement and they are shown in Figure 4C. Thulium-167 should be mostly produced through the ^168^ *Er* (*p*, 2*n*) reaction.

^168^ *Er* (*p*,*x*)^168^ *Tm*: In Figure 4D, TENDL 2021 calculations and the measured data points are in good agreement. Theoretical evaluations underestimate the production in the energy range between 11 and 14 MeV. The ^168^ *Er* (*p*, *n*) reaction should provide the major contribution to the production.

The thulium-167 cross-section uncertainties are dominated by the uncertainty of the branching ratio of its main gamma line (207.8 keV), which shows a high relative error of approximately 19% (Baglin, 2000). Considering all the numerical data are reported in Table 3 and Table 4, it would be possible to recalculate the cross sections $\left(\bar{\sigma }\right)$ with lower uncertainties once a more precise branching ratio value of thulium-167 $\left({\bar{I}}_{\gamma ,abs}\right)$ is determined:

$$\bar{\sigma }=\frac{I_{\gamma ,abs}}{{\bar{I}}_{\gamma ,abs}}\cdot \sigma$$ (3)

Moreover, the production cross sections measured in this study were also employed to derive the nuclear cross sections contributing to the production of the relevant thulium radioisotopes. A technique based on the inversion of a linear system of equations was used to achieve this objective (Braccini et al., 2022). This method necessitates the acquisition of the production cross sections of various target materials, each characterized by distinct isotopic distribution. The corresponding numerical results are described in Dellepiane et al. (2023).

### 3.2.2 Production yield measurements using the BTL

The experimental cross-section results were validated in the BTL. The experimental production yields of each relevant thulium radioisotope were compared with calculations based on nuclear cross sections of the predominant nuclear reactions within the investigated energy spectrum (6.8 MeV–18.2 MeV) (Dellepiane et al., 2023). Numerical calculations were performed considering an ideal target with the following average characteristics based on experimental measurements (39 ± 2) mg mass (0.27 ± 0.02) mm thickness and (5.1 ± 0.4) g/cm^3^ density (1-*σ* error). The following equation was adopted to evaluate the thin-target product yield (Otuka and Takács, 2015):

$$y=\frac{N_{Av}\cdot \eta }{m_{\mathrm{mol}}}\cdot \frac{1}{Z\cdot q}\int_{E_{\mathrm{out}}}^{E_{in}}\frac{\sigma \left(E_{p}\right)}{\left(-\frac{dE}{d\left(\rho x\right)}\right)\left(E_{p}\right)}dE_{p}\;\;\left(\frac{nuclei}{\mu C}\right)$$ (4)

where *Z* is the charge of the particle projectile, *q* the elementary charge, *η* the stoichiometric number of the target material, *N*_*Av*_ Avogadro’s number, *m*_*mol*_ the molecular mass of the target material, *ρ* the density of the target material, (−*dE*/*d* (*ρx*)) the mass stopping power as a function of the proton beam energy (*E*_*p*_), *E*_*in*_ the inlet proton energy at the target entrance surface, *E*_*out*_ the exiting proton energy from the target rear surface, *σ*(*E*_*p*_) the interpolated cross section based on numerical values experimentally measured as a function of the proton beam energy. The EoB thin target yield (TTY) is calculated with:

$$a\left(t_{i}\right)=\left(1-e^{-\lambda t_{i}}\right)\cdot y\;\;\left(\frac{MBq}{\mu A}\right)$$ (5)

where *t*_*i*_ is the irradiation time. For the evaluation of the production yield, the physical thin target yield was adopted and calculated with the following equation:

$$\alpha_{\mathrm{phys}}={\left.\frac{da\left(t_{i}\right)}{dt_{i}}\right|}_{t_{i}=0}=\lambda \cdot y\;\;\left(\frac{MBq}{\mu Ah}\right)$$ (6)

which could be considered as a figure of merit only if the product *λ* ⋅ *t*_*i*_ ≪ 1. In addition, the radionuclidic purity as a function of the proton energy (*E*_*p*_) was calculated as follows:

$$P\left(E_{p}\right)=\frac{\alpha_{\mathrm{phys,Tm-167}}\left(E_{p}\right)}{\sum_{X=165}^{168}\left(\alpha_{\mathrm{phys,Tm-X}}\left(E_{p}\right)\right)}$$ (7)

**FIGURE 5:**
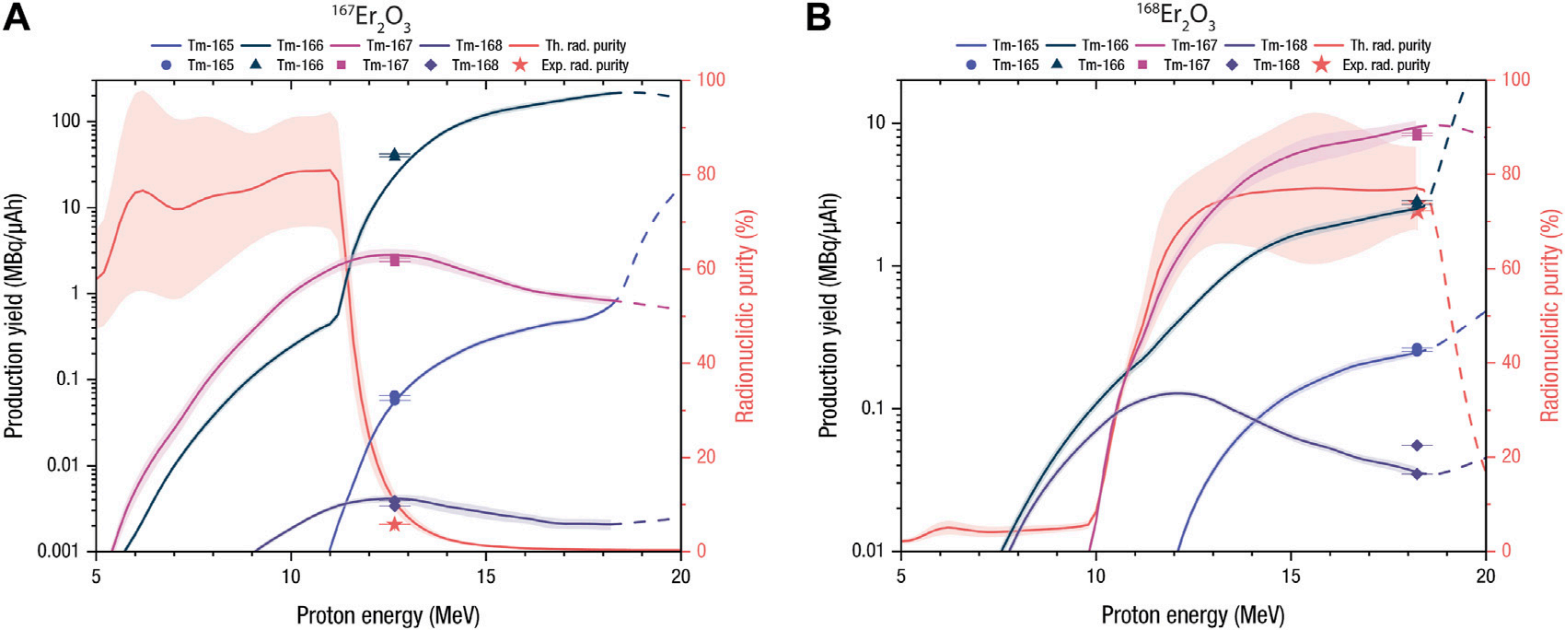
Production yields of the relevant thulium radioisotopes (left-handed scale) and radionuclidic purity at EoB (right-handed scale) based on our experimentally determined cross sections as functions of the target-inlet proton energy using **(A)** ^167^ *Er*_2_ *O*_3_ and **(B)** ^168^ *Er*_2_ *O*_3_ as target materials. The bands correspond to the maximum and minimum yield derived from the measured cross sections. The solid lines correspond to production yield evaluations based on experimental cross sections. The dashed lines represent theoretical production yields calculated using TENDL 2021 cross sections due to the lack of experimental numerical values.

The exiting proton energies were calculated with SRIM (Ziegler et al., 2010). Figure 5 shows that the optimum proton energies are 12.7 MeV and 18.2 MeV for the irradiations of ^167^Er_2_O_3_ and ^168^Er_2_O_3_, respectively, maximizing thulium-167 production. However, analyzing the production yields for ^168^Er_2_O_3_, it can be supposed that higher proton energies might lead to higher values. The experimental results are plotted in Figures 5A,B for ^167^Er_2_O_3_ and ^168^Er_2_O_3_, respectively. The numerical values are reported in Table 5 together with the corresponding evaluated production yield based on the previously measured cross sections, irradiation parameters and target masses. Predictions (Eq. 6) and experimental results are on average in good agreement. The small differences might be explained by the numerical calculation method. This approach solely studied a single target configuration, whereas it was experimentally observed that mass and thickness may vary starkly during target preparation. This variation also influences target density, which is derived by approximating the target volume to a cylinder. The production yield of thulium-166 evaluated with the measured cross sections using ^167^Er_2_O_3_ as target material deviates significantly from experimental measurements. This deviation might be caused by considering the uncertainty of the exiting proton energies. From simulations it was observed that this energy can be found mostly in the energy range of (9–10) MeV, which corresponds to the region where the production cross section exhibits a steep increase. In this case, the uncertainties on target characteristics, especially target density, may play a significant role, since they highly affect the calculated exiting proton energies. Table 5 also reports the radionuclidic purities evaluated at EoB and after a cooling time of 5 days. This choice is particularly beneficial in the case of enriched erbium-167 oxide, where the large thulium-166 EoB activity might be neglected.

**TABLE 5:** Experimental production yields of the relevant thulium isotopes using targets with average mass of 40 mg in the BTL. Production yield calculations based on our measured cross sections (Eq. 6) are reported below the experimental values between round [...] brackets and in italic. Experimental radionuclidic purity is also reported with its prediction for each target at EoB and after 5 days of cooling time. Inlet and exiting proton energies, irradiation time, integrated proton current and target masses are also tabulated.

	^167^Er_2_O_3_		^168^Er_2_O_3_	
	T1 (MBq/*μ*Ah)	T2 (MBq/*μ*Ah)	T1 (MBq/*μ*Ah)	T2 (MBq/*μ*Ah)
^165^Tm	0.057 ± 0.005	0.066 ± 0.006	0.25 ± 0.02	0.27 ± 0.03
	*(0.056)*	*(0.055)*	*(0.25)*	*(0.25)*
^166^Tm	39 ± 3	42 ± 4	2.9 ± 0.2	2.7 ± 0.2
	*(24)*	*(23)*	*(2.5)*	*(2.5)*
^167^Tm	2.3 ± 0.5	2.6 ± 0.5	8.2 ± 1.6	8.5 ± 1.7
	*(2.7)*	*(2.9)*	*(9.4)*	*(9.4)*
^168^Tm	0.0034 ± 0.0002	0.0039 ± 0.0003	0.035 ± 0.005	0.06 ± 0.02
	*(0.0040)*	*(0.0042)*	*(0.036)*	*(0.036)*
Rad. Purity (t_*cool*_ = 0) (%)	5.7 ± 1.2	5.8 ± 1.2	72.2 ± 4.1	73.9 ± 3.9
	*(10.3)*	*(10.9)*	*(77.1)*	*(77.1)*
Rad. Purity (t_*cool*_ = 5 d) (%)	99.5 ± 0.1	99.5 ± 0.1	99.1 ± 0.2	98.8 ± 0.4
	*(99.6)*	*(99.6)*	*(99.2)*	*(99.2)*
Irradiation time h)	0.22	0.22	0.24	0.31
Int. current (*μ*C)	2.7 ± 0.1	2.7 ± 0.1	3.0 ± 0.2	3.5 ± 0.2
E_*p*,*in*_ (MeV)	12.7 ± 0.4		18.2 ± 0.4	
E_*p*,*out*_ (MeV)	9.9 ± 0.5	9.7 ± 0.5	16.1 ± 0.4	
Target mass (mg)	37.8	40.4	39.4	39.7

### 3.3 Production yield measurements at the Paul Scherrer Institute

At PSI, the project involved the assessment of thulium-167 production yields using five different target materials (^167^Er_2_O_3_, ^168^Er_2_O_3_, ^*nat*^Tm_2_O_3_, ^*nat*^Yb_2_O_3_, ^171^Yb_2_O_3_). The production tests data are summarized in Table 6, together with average beam energy, average target masses, and irradiation time. In the table, three relevant quantities are reported: the average thulium-167 activity, the experimental EoB thin-target yields (Eq. (5)), and the radionuclidic purities. The latter was evaluated after a cooling time of 5 days from EoB to allow for the decay of short-lived thulium radionuclides. The highest activities were obtained with ^168^ *Er*_2_ *O*_3_ and ^171^ *Yb*_2_ *O*_3_. The first target material showed high production yields when irradiated with rather low proton energies (18.7 MeV–24.6 MeV). However, the maximum produced activity, unexpectedly, was found to be at 22.8 MeV. Based on the findings derived from the proton irradiation of natural erbium as documented by Tárkányi et al. (2008), it is reasonable to draw certain conclusions. In the explored energy range, the predominant nuclear reaction is the ^168^ *Er* (*p*,2*n*)^167^ *Tm* reaction, irrespective of isotopic distribution. The same reaction mostly contributes in the production of thulium-167 for proton-irradiated PSI targets of enriched erbium-168 oxide. Therefore, it is reasonable to qualitatively compare the theoretical production yield trend calculated using the production cross sections of Tárkányi et al. (2008). As a result, the predictions show that the maximal production should be at around 19.5 MeV, in contrast with production tests performed at IP2. In relation to ytterbium oxides, initial proton irradiation of only natural ytterbium using two proton energies (34.0 MeV and 40.4 MeV) yielded relatively low levels of activity. A new target holder was subsequently built to achieve higher proton energies at the IP2 target station. Therefore, additional production tests were carried out to validate the results obtained by Tárkányi et al. (2009). Afterward, enriched ytterbium-171 oxide was also selected in order to significantly increase thulium-167 production yield. Enriched ytterbium-171 oxide seemed to be a promising candidate for thulium-167 production using high-energy proton beams. Experimental results for 8-h irradiations reported an EoB activity of thulium-167 of about 420 MBq on average. Production routes using ytterbium oxides as target material are limited by the production saturation due to the short half-life of the direct product, lutetium-167 (*t*_1/2_ = 51.5 *m*), with respect to the irradiation time. On the other hand, predictions for the same irradiation period show that proton irradiations of enriched erbium-168 oxide using a 23-MeV proton beam could yield 1 GBq of thulium-167 activity.

**TABLE 6:** Average EoB activities of thulium-167, EoB TTYs of thulium-167 (Equation 5) and radionuclidic purities evaluated after 5 days from EoB measured at PSI. Upper limits of the radionuclidic purity are reported whenever thulium-168 was only detected. At least two targets were irradiated for each configuration. In the table, it is also reported the average target mass, irradiation time and average proton beam energy (error 1-*σ*), whose values were calculated with BDSIM. The target thickness is approximately 270 *μ*m for each target.

Target material	**E**_*p*_ * (MeV)	**Target mass** (mg)	**Irradiation time** (min)	**Ave.** ^167^ **Tm A**_*EoB*_ (MBq)	EoB TTY** (MBq/*μ*A)	**Rad. Purity** (**t**_*cool*_ = 5*d* **)** (%)
^167^Er_2_O_3_	10.6 ± 2.5	40.5	30	3.2 ± 0.2	0.17 ± 0.01	< 99.64 ± 0.02
	12.3 ± 2.3	39.0	30	5.9 ± 0.8	0.32 ± 0.05	< 99.93 ± 0.01
	**13.8 ± 2.1**	42.5	5	2.4 ± 0.3	0.13 ± 0.02	99.71 ± 0.04
	**13.8 ± 2.1**	102.0	5	3.0 ± 0.4	0.12 ± 0.02	99.65 ± 0.05
^168^Er_2_O_3_	18.7 ± 1.9	38.8	5	8.2 ± 0.8	0.44 ± 0.04	98.7 ± 0.1
	18.7 ± 1.9	41.0	30	50 ± 7	2.7 ± 0.4	< 98.4 ± 0.2
	18.7 ± 1.9	98.0	5	15.9 ± 3.0	0.64 ± 0.12	< 98.2 ± 0.3
	18.7 ± 1.9	39.0	120	226 ± 31	12.0 ± 1.7	< 98.7 ± 0.2
	**22.8 ± 1.8**	40.0	5	8.9 ± 1.3	0.46 ± 0.07	< 99.63 ± 0.07
	**22.8 ± 1.8**	39.0	30	73 ± 11	3.8 ± 0.5	< 99.44 ± 0.08
	24.6 ± 1.8	41.0	5	9.7 ± 1.3	0.50 ± 0.07	< 99.41 ± 0.08
^*nat*^Yb_2_O_3_	34.0 ± 1.8	41.5	30	Not detected	Not detected	Not detected
	40.4 ± 2.2	41.0	5	0.58 ± 0.05	0.028 ± 0.002	<100
	40.4 ± 2.2	41.3	30	2.7 ± 0.4	0.13 ± 0.02	<100
	51.1 ± 2.7	42.0	5	2.0 ± 0.3	0.09 ± 0.01	<100
	**62.6 ± 2.7**	41.0	5	5.8 ± 0.8	0.27 ± 0.04	<100
^171^Yb_2_O_3_	**51.1 ± 2.7**	40.0	5	8.6 ± 1.2	0.40 ± 0.06	<100
	**51.1 ± 2.7**	40.0	120	238 ± 34	11.1 ± 1.6	< 99.95 ± 0.01
	**51.1 ± 2.7**	39.0	480	421 ± 42	19.7 ± 2.0	< 99.973 ± 0.003
	53.0 ± 2.7	41.0	5	10.2 ± 1.2	0.48 ± 0.05	< 99.95 ± 0.01
^*nat*^Tm_2_O_3_	24.6 ± 1.8	41.0	10	10.3 ± 0.6	0.53 ± 0.03	< 97.9 ± 0.1
	26.4 ± 1.7	38.5	5	11.9 ± 1.6	0.61 ± 0.08	< 98.5 ± 0.2
	**28.1 ± 1.7**	40.0	10	26.1 ± 1.1	1.33 ± 0.06	< 98.45 ± 0.07

*The optimum proton energy is highlighted in bold.

**Note: the thin target yield (TTY) is dependent on the irradiation time.

The radionuclidic purity was also evaluated for each target material. A cooling time of 5 days was selected for its assessment to neglect the contribution of thulium-166, while minimizing thulium-165 content. The first was never detected due to long cooling time. High activities of longer-lived radionuclides (mainly thulium-167 and thulium-168) prevented performing gamma-ray spectrometry before its activity was below the minimum detectable activity (MDA). On the other hand, thulium-165 was occasionally measured due to low production and long cooling periods. For this reason, upper limits of the radionuclidic purity are reported in Table 6 whenever thulium-168 was solely detected. The most relevant radionuclidic impurity is represented by thulium-168 due to its long half-life (93.1 d). The irradiations of enriched erbium-168 oxide exhibited a larger production of thulium-168 than enriched erbium-167 oxide. This difference is mainly due to the increase of the probability of the ^168^ *Er* (*p*,*n*)^168^ *Tm* reaction and due to the suppression of the ^167^ *Er* (*p*,*γ*)^168^ *Tm* reaction. Focusing on enriched erbium-168 oxide, irradiations with higher proton energies might have enabled a larger thulium-165 production through the ^167^ *Er* (*p*, 3*n*) reaction, despite a lower enrichment level of erbium-167.

The decision to investigate indirect production routes (Tm_2_O_3_ and Yb_2_O_3_) was also dictated by the elimination of a production channel for thulium-168 due to the presence of stable ytterbium-168. As a matter of fact, the highest radionuclidic purity was observed using ^171^ *Yb*_2_ *O*_3_ as target material exploiting the high proton energies accessible at the IP2 target station. The sole thulium-168 was detected among thulium radioisotopes due to long cooling periods. Furthermore, only prolonged irradiation periods (*t*_*irr*_ ≥ 1 *h*) enabled the production of a sufficient activity of this radionuclide, thereby enabling its detection through gamma-ray spectrometry. Consequently, the irradiations of natural ytterbium oxide showed a 100% radionuclidic purity due to short irradiation periods.

A crucial aspect might be represented by the possibility of having the stable thulium-169 in the final product. This isotope originates from the decay chain of the isobars with mass number 169 (Lu169→Yb169→Tm169) . Therefore, in order to obtain a *carrier-free* final product, a chemical separation should be performed soon after EoB. However, previous studies (Gracheva et al., 2019; Favaretto et al., 2021) showed that it is impractical to chemically separate neighboring lanthanides in few hours or minutes, preventing the separation of lutetium-167 and ytterbium-167 due to their short half-lives. As a consequence, a suitable cooling time should be considered to let thulium-167 accumulate before proceeding with the chemical separation. Based on theoretical calculations and experimental EoB activity of lutetium-169, it is reasonable to neglect thulium-169 content because of the slow decay of ytterbium-169 (*t*_1/2_ = 32.018 *d*). By solving the Bateman equations considering an 8-h irradiation, it is possible to determine: i) a suitable cooling time, approximately 7.5 h, and ii) thulium-169 content (mTm169≈0.04ng) , which should not affect the radiolabeling step considering the larger amount of thulium-167 (mTm167≈130ng) .

A slight difference in the enriched erbium oxide data was observed when comparing the production yield results measured at PSI and at the Bern medical cyclotron laboratory. For example, by employing a comparable beam energy for enriched erbium-168 oxide, a production yield of 7.0 MBq/*μ*Ah was obtained at PSI, in contrast to the 8.3 MBq/*μ*Ah measured at the Bern medical cyclotron laboratory. The discrepancy might be attributed to an imperfect control over the parameters of the proton beam, e.g., the proton current, when impinging on the target surface. Future work will be devoted to experimentally assess these beam characteristics and improve the beam positioning at the IP2 target station. At the current stage, simulation results are used to evaluate the proton current impinging onto the target at the IP2 target station projecting the 50-*μ*A current measured inside the vacuum chamber. These objectives will be achieved by exploiting monitor cross sections and by implementing a new beam monitor system, respectively. The latter will consist of four sets of thermocouples installed prior to the aluminum vacuum window.

In conclusion, the proton irradiation of ^168^Er_2_O_3_ could potentially yield approximately an activity of 1 GBq with a radionuclidic purity of < 99.5% (*t*_*cool*_ = 5 *d*, 0.5% of activity impurity assigned to thulium-168). This can be achieved by bombarding a target measuring 40 mg in mass and 6 mm in diameter, under the following irradiation conditions: 8 h irradiation time, 22.8 MeV proton beam energy, 19.3 *μ*A (from simulations) proton current. Unfortunately, large production of thulium-167 with ^171^Yb_2_O_3_ is impractical due to the saturation of its short-lived parent nuclide lutetium-167 (*t*_1/2_ = 51.5 *m*). However, this production route is still appealing for its high level of radionuclidic purity ( <99.95 %, *t*_*cool*_ = 5 *d*, only thulium-168 was detected), although the highest registered thulium-167 activity was of 420 MBq after 8 h irradiation time. This result was obtained irradiating a 40-mg, 6-mm-diameter target disk using a proton beam with the following characteristics: 51.1 MeV proton beam energy, 21.4 *μ*A (from simulations) proton current.

## 4 Conclusion and outlook

In this study, the production of the medically relevant radionuclide thulium-167 was investigated using both medical (18 MeV) and research (72 MeV) cyclotrons. In general, the comparison between experimental results and theoretical predictions of TENDL 2021 exhibited a good agreement. Experimental cross-section data were validated by performing production yield measurements. The production yields of 2.4 MBq/*μ*Ah (*E*_*p*_ = 12.7 *MeV*) and 8.3 MBq/*μ*Ah (*E*_*p*_ = 18.2 *MeV*) were measured for ^167^ *Er*_2_ *O*_3_ and ^168^ *Er*_2_ *O*_3_, respectively. However, the large uncertainty of the emission intensity of the 208-keV gamma ray (*u*_*rel*_ = 19%) significantly affects the precision of thulium-167 cross-section data. To achieve more accurate cross-section measurements, future metrological research will be addressed to improve the precision and accuracy of 208-keV gamma line of thulium-167.

The systematic investigation of the production yields using various target materials (^167^Er_2_O_3_, ^168^Er_2_O_3_, ^*nat*^Tm_2_O_3_, ^*nat*^Yb_2_O_3_, ^171^Yb_2_O_3_) for the thulium-167 production allowed to determine the most promising production route for medical applications. ^168^Er_2_O_3_ stood out as the most favorable candidate target material for the production of thulium-167 on a large scale with a radionuclidic purity of < 99.5% (*t*_*cool*_ = 5 *d*). An efficient and robust radiochemical separation technique will be devised to pursue high specific activities of thulium-167 in view of its future medical applications. Further studies should also be conducted to ascertain whether the radionuclidic impurity levels, specifically thulium-168, could potentially impose constraints in relation to radiation protection and patient dosimetry.

This study and the following steps will enable subsequent preclinical investigations aimed at exploring the eventual use of this radionuclide in therapy and imaging.

## Data Availability

The original contributions presented in the study are included in the article/Supplementary Material, further inquiries can be directed to the corresponding author.
