# Supplementary material for: Study of thulium-167 cyclotron production: a potential medically-relevant radionuclide
Source: Front Chem. 2023 Oct 19;11:1288588. doi: 10.3389/fchem.2023.1288588 (PMC10620610; doi:10.3389/fchem.2023.1288588)
Supplement: Supplementary file 1 [file DataSheet1.PDF]

Supplementary Material

1 SUPPLEMENTARY TABLES AND FIGURES

1.1 Tables

Table S1. Simulated proton energies, uncertainties (evaluated at 1-σ interval), and proton currents for each niobium degrader-foil and water cooling thickness available at IP2 target station of PSI. A horizontal line divides the data to distinguish between the two configurations represented by Figures S1a and S1b, wherein one configuration includes niobium degraders while the other does not. The simulations were performed with BDSIM (Nevay et al., 2020).

Thickness (mm)		E_p (MeV)	$u_{abs}(E_p)$ (MeV)	I_p (μA)	
Nb	H_2O			6 mm diameter	10 mm diameter
3.5	20.5	8.9	2.6	17.7	23.3
3.4	20.6	10.6	2.5	18.3	24.0
3.3	20.7	12.3	2.3	18.5	24.2
3.2	20.8	13.8	2.1	18.7	24.3
3.1	20.9	15.1	2.0	18.7	24.5
3.0	21.0	16.4	2.0	18.7	24.6
2.8	21.2	18.7	1.9	18.9	24.7
2.4	21.6	22.8	1.8	19.3	25.0
2.2	21.8	24.6	1.8	19.3	25.1
2.0	22.0	26.4	1.7	19.5	25.3
1.8	22.2	28.1	1.7	19.7	25.4
1.0	23.0	34.0	1.8	20.1	25.7
	24.0	40.4	2.2	20.7	26.1
	16.0	51.1	2.7	21.4	27.0
	14.5	53.0	2.7	21.4	27.0
	13.0	54.8	2.7	21.6	27.1
	11.5	56.5	2.7	21.7	27.1
	10.0	58.2	2.8	21.7	27.3
	8.5	59.9	2.8	21.9	27.4
	7.0	61.5	2.8	21.9	27.4
	6.0	62.6	2.7	22.0	27.4

Table S2. Isotopic distributions of enriched erbium oxides used for cross-section measurements. Experiments were conducted using ICP-MS to validate the numerical values reported on the certificate of analysis (CoA) (ISOFLEX USA (Isoflex, 2023)).

	$^{167}Er_2O_3$				$^{168}Er_2O_3$	
	BATCH 1		BATCH 2		BATCH 1	
	Exp. (%)	CoA (%)	Exp. (%)	CoA (%)	Exp. (%)	CoA (%)
^{162}Er	4.3E-4 (15)	< 0.01	3.3E-3 (12)	< 0.01	8.7E-4 (43)	-
^{164}Er	9.0E-3 (3)	0.01	4.7E-2 (4)	0.01	2.0E-2 (45)	-
^{166}Er	0.986 (8)	0.96	1.77 (8)	0.96	0.365 (6)	0.37
^{167}Er	96.2 (5)	96.3 (1)	94.4 (3)	96.3 (1)	2.65 (38)	0.72
^{168}Er	2.60 (2)	2.57	3.24 (5)	2.57	96.3 (10)	98.3
^{170}Er	0.197 (4)	0.16	0.57 (4)	0.16	0.643 (5)	0.61

Table S3. Isotopic distributions of natural and enriched ytterbium-171 oxides. The latter was experimentally assessed by means of ICP-MS and compared to the numerical values of the certificate of analysis (CoA) (ISOFLEX USA (Isoflex, 2023)). Natural abundances were obtained from Meija et al. (2016).

	$^{171}Yb_2O_3$		$^{nat}Yb_2O_3$
	Exp. (%)	CoA (%)	Meija et al. (2016) (%)
^{168}Yb	8.7E-4 (6)	< 0.01	0.12
^{170}Yb	8.2E-2 (2)	0.08	2.98
^{171}Yb	95.5 (27)	95.5 (2)	14.09
^{172}Yb	3.63 (11)	3.63	21.69
^{173}Yb	0.301 (9)	0.31	16.10
^{174}Yb	0.36 (1)	0.39	32.03
^{176}Yb	0.082 (2)	0.09	13.00

1.2 Figures

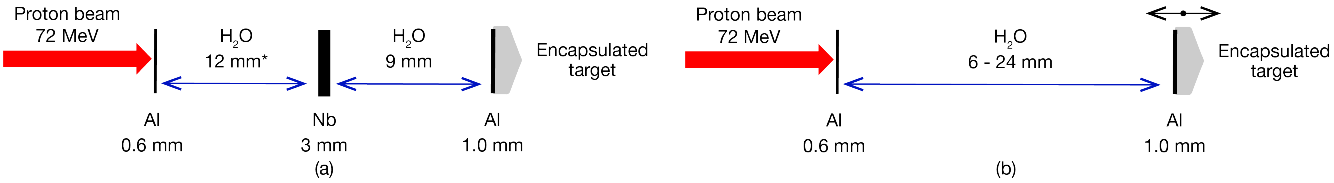

Figure S1. Simplified target station structures for **(a)** low and **(b)** high proton energy range to simulate energy degradation. Dimensions refer to thicknesses of the layers. **(a)** Water layer of 12 mm (*) varies according to the thickness of the interchangeable niobium degraders (from Grundler et al. (2020)). **(b)** The target-vacuum window distance may be changed according to the desired proton energy by moving the target capsule. The position of aluminum entrance vacuum window (left) is fixed.

REFERENCES

Grundler, P. V., Eichler, R., Talip, Z., Schubiger, P. A., Schibli, R., and van der Meulen, N. P. (2020). The metamorphosis of radionuclide production and development at Paul Scherrer Institute. *Chimia* 74, 968–975. doi:10.2533/CHIMIA.2020.968

Isoflex (2023). ISOFLEX USA

Meija, J., Coplen, T. B., Berglund, M., Brand, W. A., De Bièvre, P., Gröning, M., et al. (2016). Isotopic compositions of the elements 2013 (IUPAC Technical Report). *Pure and Applied Chemistry* 88, 293–306. doi:10.1515/pac-2015-0503

Nevay, L. J., Boogert, S. T., Snuverink, J., Abramov, A., Deacon, L. C., Garcia-Morales, H., et al. (2020). BDSIM: An accelerator tracking code with particle–matter interactions. *Computer Physics Communications* 252, 107200. doi:10.1016/J.CPC.2020.107200
